# Hypoxia, Blackwater and Fish Kills: Experimental Lethal Oxygen Thresholds in Juvenile Predatory Lowland River Fishes

**DOI:** 10.1371/journal.pone.0094524

**Published:** 2014-04-11

**Authors:** Kade Small, R. Keller Kopf, Robyn J. Watts, Julia Howitt

**Affiliations:** 1 School of Environmental Sciences, Charles Sturt University, Albury, New South Wales, Australia; 2 Institute for Land, Water and Society, Charles Sturt University, Albury, New South Wales, Australia; 3 School of Agricultural and Wine Sciences, Charles Sturt University, Wagga Wagga, New South Wales, Australia; The Evergreen State College, United States of America

## Abstract

Hypoxia represents a growing threat to biodiversity in freshwater ecosystems. Here, aquatic surface respiration (ASR) and oxygen thresholds required for survival in freshwater and simulated blackwater are evaluated for four lowland river fishes native to the Murray-Darling Basin (MDB), Australia. Juvenile stages of predatory species including golden perch *Macquaria ambigua*, silver perch *Bidyanus bidyanus*, Murray cod *Maccullochella peelii*, and eel-tailed catfish *Tandanus tandanus* were exposed to experimental conditions of nitrogen-induced hypoxia in freshwater and hypoxic blackwater simulations using dried river red gum *Eucalyptus camaldulensis* leaf litter. Australia's largest freshwater fish, *M. peelii*, was the most sensitive to hypoxia but given that we evaluated tolerances of juveniles (0.99±0.04 g; mean mass ±SE), the low tolerance of this species could not be attributed to its large maximum attainable body mass (>100,000 g). Concentrations of dissolved oxygen causing 50% mortality (LC_50_) in freshwater ranged from 0.25±0.06 mg l^−1^ in *T. tandanus* to 1.58±0.01 mg l^−1^ in *M. peelii* over 48 h at 25–26°C. Logistic models predicted that first mortalities may start at oxygen concentrations ranging from 2.4 mg l^−1^ to 3.1 mg l^−1^ in *T. tandanus* and *M. peelii* respectively within blackwater simulations. Aquatic surface respiration preceded mortality and this behaviour is documented here for the first time in juveniles of all four species. Despite the natural occurrence of hypoxia and blackwater events in lowland rivers of the MDB, juvenile stages of these large-bodied predators are vulnerable to mortality induced by low oxygen concentration and water chemistry changes associated with the decomposition of organic material. Given the extent of natural flow regime alteration and climate change predictions of rising temperatures and more severe drought and flooding, acute episodes of hypoxia may represent an underappreciated risk to riverine fish communities.

## Introduction

Hypoxia and blackwater events occur in lowland rivers and wetlands world-wide and can be a natural phenomenon associated with rising temperature, floodplain inundation and the subsequent decomposition of organic material [1], [2], [3]. Excess loads of allocthonous organic material, nutrients or rising temperature can stimulate acute episodes of hypoxia and water chemistry changes. Hypoxic blackwater events are usually characterized by elevated dissolved organic carbon (DOC) concentrations, tea coloured water, low dissolved oxygen concentration [2], [3] and reduced pH associated with the release of organic acids and polyphenols from terrestrial organic matter; some of which may be toxic [4]. Blackwater rivers, as opposed to sporadic episodes of hypoxic blackwater, are the result of a consistent annual cycle of floodplain inundation [5].

**Figure 1:**
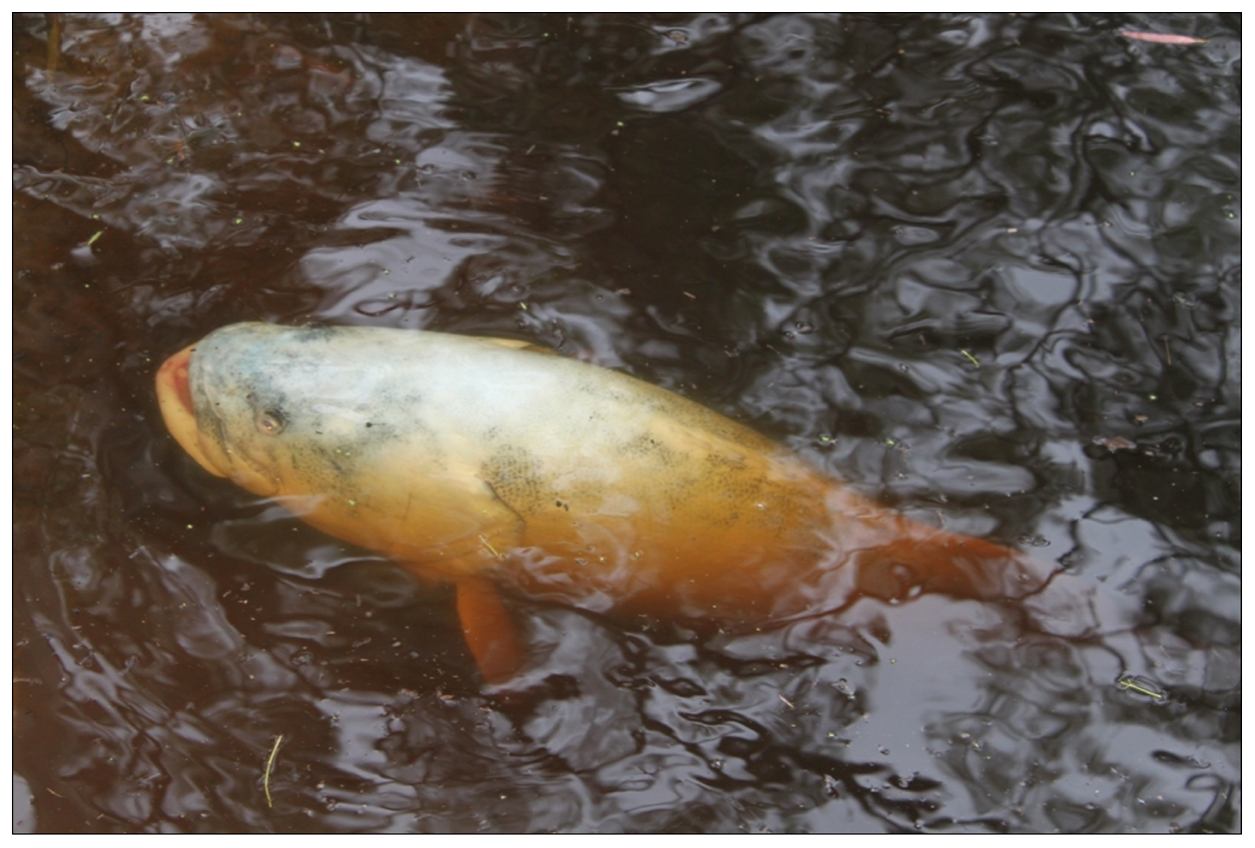
A severe hypoxic blackwater event resulted in extensive fish kills in the Murray-Darling Basin, Australia in 2010/11. Photograph shows a Murray cod *Maccullochella peelii* (Mitchell) appearing to perform aquatic surface respiration (ASR) during the hypoxic blackwater event in the Edward River near Moulamein, New South Wales. Printed under a CC BY license with permission from L. Pearce, 2011.

Fish inhabiting rivers and wetlands where hypoxia occurs may be adapted to tolerate low dissolved oxygen concentrations and variable water quality using a variety of mechanisms ranging from air-breathing to behavioural avoidance [6]. Using behaviour referred to as aquatic surface respiration (ASR), many species of fish will rise to the surface when exposed to hypoxia, in an attempt to extract oxygen from the thin surface layer of water that is contact with the atmosphere (Figure 1). Severe, widespread, or prolonged low-oxygen events may, however, lead to sub-lethal effects and mortalities [2], [4], [7], [8]. In the United States hypoxia is estimated to be the proximate cause of 5.3% of fish kills [7], although low oxygen may be the ultimate cause of death in some mortalities ascribed to eutrophication, pollution or extreme climate events such as flooding and drought. Developing an understanding of low dissolved oxygen tolerances and sub-lethal effects on aquatic animals will be essential in developing natural resource management guidelines to mitigate the effects of human-induced hypoxia.

Given the natural occurrence of drought, flooding and blackwater events in lowland rivers of the Murray-Darling Basin (MDB) in south-eastern Australia [1], , it is reasonable to assume that native fish in this ecosystem would be well adapted to acute hypoxia and high levels of DOC. However, after 10 years of drought, since the year 2000, the MDB experienced large-scale floods followed by hypoxic blackwater events which caused wide-spread fish kills [2], [8]. Estimates of the total number of fish mortalities and species composition following this event have not been published but anecdotal and scientific reports on fish kills in the MDB have tended to focus on large-bodied recreationally important species [7], [8], [9]. It remains unclear whether large-bodied species are disproportionately reported in fish kills because of low tolerances to hypoxic blackwater or because small-bodied species are under-reported. Previous research on Australian freshwater fishes suggests a relatively high degree of hypoxia, DOC and toxic leachate tolerance amongst small-bodied species [10], [11], [12] but very little research has been undertaken on the hypoxic blackwater tolerances of large-bodied lowland fishes [4], [13], [14] most commonly reported in fish kills [7], [9].

This study aimed to quantify low dissolved oxygen (DO) blackwater tolerances in juvenile stages of four lowland river fishes native to the MDB, Australia. The species examined included juvenile stages of: Murray cod *Maccullochella peelii* (Mitchell), golden perch *Macquaria ambigua* (Richardson), silver perch *Bidyanus bidyanus* (Mitchell) and eel-tailed catfish *Tandanus tandanus* (Mitchell). As adults, these species are top predators and consumers in lowland river ecosystems of the MDB and are important to recreational fisheries. The objectives of this research were to: 1) determine if there are species-specific differences in lethal oxygen concentrations and ASR responses and 2) determine whether lethal oxygen concentrations and ASR responses are influenced by increasing DOC concentrations within simulated blackwater.

## Materials and Methods

### Experimental design

Between two-hundred and two-hundred and fifty juveniles each of four species (Table 1) were sourced from Urarah Fisheries (*M. peelii*), New South Wales Department of Primary Industries – Narrandera (*M. ambigua*), Murray Cod Hatcheries (*T. tandanus*) and Silverwater Fisheries (*B. bidyanus*). All hatcheries used wild brood-stock. Juveniles were used to develop low oxygen thresholds because of conservation restrictions concerning the death of large numbers of threatened wild-caught adults and number of years required for hatchery fish to reach maturity. All fish were in the juvenile stage of development and approximately 6 to 14 months of age, ranging from 30 mm to 88 mm standard length (Table 1). Differences in hatch date resulted in inter-specific variation in body size but there were no significant differences in mean mass or length within species' treatments. Fish were transported within one day from the hatcheries to a temperature controlled room maintained at 25–26°C for the duration of experimentation.

**Table 1:** Mean body size measurements and water quality parameters for hypoxia experiments conducted on Murray-Darling Basin fishes.

	Hypoxic freshwater						Simulated hypoxic blackwater					
Species	*n*	Length (mm)	Mass (g)	Temp. (°C)	pH range	DO (mg l^−1^) range	*n*	Length (mm)	Mass (g)	Temp. (°C)	pH range	DO (mg l^−1^) range
Murray cod *Maccullochella peelii*	80	50.42 (0.68)	0.99 (0.04)	25.63 (0.15)	7.69–8.65	0.16–8.24	60	51.34 (0.80)	1.01 (0.02)	25.02 (0.07)	6.61–7.80	0.17–8.26
Golden perch *Macquaria ambigua*	80	36.06 (0.88)	0.45 (0.03)	25.49 (0.09)	7.67–8.06	0.07–8.14	60	37.38 (0.43)	0.48 (0.02)	24.51 (0.11)	6.77–7.51	0.06–8.2
Eel-tailed catfish *Tandanus tandanus*	80	83.90 (1.64)	3.67 (0.19)	26.35 (0.22)	7.23–8.74	0.09–8.68	60	83.45 (0.92)	3.61 (0.10)	25.73 (0.12)	6.49–7.11	0.08–8.5
Silver perch *Bidyanus bidyanus*	70	35.4 (0.69)	0.40 (0.02)	26.22 (0.14)	7.41–8.55	0.09–8.67	50	35.98 (0.47)	0.43 (0.02)	26.71 (0.09)	6.05–7.22	0.09–8.3

Fish were exposed to 48 h lethal dissolved oxygen (DO) in hypoxic freshwater and simulated hypoxic blackwater. Mean ±(SE).

Each species was acclimated to a constant temperature of 25–26°C for between 9 to 15 days prior to experimentation. Lights were programmed for a 12 h light and dark cycle and fish were fed blood worms twice daily except during experimentation. Species were acclimated separately in 750 l holding tanks equipped with a UV light filter and a recirculating bio-ball filtration system that maintained oxygen levels above 7 mg l^−1^. All experiments were undertaken in glass aquaria, 23 cm (L)×23 cm (W)×45 cm (H), custom built to achieve minimal surface area and limit oxygen diffusion with the atmosphere. A static flow during all experiments was utilized to reduce variation in DO concentration. Prior to each experiment oxygen sensors (YSI rapid pulse electrochemical sensor; 6-series) were calibrated against temperature-specific oxygen saturated distilled water.

Hypoxia tolerance of each species was quantified using median lethal oxygen concentration (LC_50_) and aquatic surface respiration (ASR_50_) experiments conducted in accordance with international guidelines for acute toxicity testing [15] in: 1) hypoxic freshwater (no dissolved organic carbon/leaf litter) and 2) simulated hypoxic blackwater created using dried leaf litter. Experiments were carried out with permission from a Charles Sturt University Animal Care and Ethics (ACEC) committee protocol; number 11/018. The acute toxicity guidelines and ACEC protocol aimed to minimize the number of animals used whilst generating LC_50_ and ASR_50_ estimates under hypoxic blackwater and hypoxic freshwater experimental conditions for each species.

### Hypoxic freshwater

Seven oxygen treatments, each containing 10 fish replicated per aquaria, were maintained during hypoxic freshwater LC_50_ and ASR_50_ experiments for each species. Treatments consisted of DO concentrations of 0.1 mg l^−1^, 0.7 mg l^−1^, 1.3 mg l^−1^, 1.9 mg l^−1^, 2.5 mg l^−1^, 3.1 mg l^−1^ and a control (8 mg l^−1^) that were maintained at 25–26°C for 48 h. The experimental temperature of 25–26°C was warmer than the majority of hypoxic blackwater events documented in the MDB [1]–[3], although it does represent the mid-point of the range of temperatures recorded during the 2005 blackwater event in the Barmah Forest [3]. Given that oxygen metabolism in fish increases with temperature, therefore, the hypoxia tolerances estimated here were intended to provide a precautionary estimate for application by natural resource managers. Previous studies assessing hypoxia on small-bodied fish species of the MDB were conducted at 25°C, as it represents a temperature commonly reached in summer in billabongs in the MDB [12] and the main river channel of the Murray River at Barmah [3]. Dissolved oxygen was regulated using nitrogen (N_2_) or atmospheric air. Hypoxic freshwater experiments contained no leaf litter and therefore little or no DOC. Atmospheric air and N_2_ were regulated via pressure valves and separate air stones placed at the bottom of experimental tanks filled with 17.5 l of holding tank water. Experimental DO concentrations were maintained at all times and treatments to within 0.3 mg l^−1^ of the pre-determined oxygen concentration or were excluded from the analyses.

Ten fish within each aquaria served as replicates for each species-oxygen treatment combination and one randomly selected DO treatment was repeated. The experimental design resulted in 20 replicates for the repeated treatment and 10 replicates for other species-oxygen treatment combinations. Repeated treatments included DO concentrations of 3.1 mg l^−1^ for *M. ambigua*, 0.7 mg l^−1^ for *T. tandanus* and 2.5 mg l^−1^ for *M. peelii*. A more rigorous experimental design would have replicated each aquaria of 10 fish at least three times, although the total number of fish used here ultimately represented trade-offs among statistical power, animal ethics and limitations concerning species conservation status and project resources. The present design provided sufficient statistically power for all four species but individual oxygen-species treatments (n = 10) were largely unreplicated at the tank level.

The highest DO treatment concentration, used for all species, was pre-determined from range finding trials conducted in accordance with the international guidelines for acute toxicity testing [15]. In range finding trials oxygen levels were reduced in 10 increments from 8 mg l^−1^ down to 0.5 mg l^−1^ over a two hour period. This period of time allows for comparisons with other similar studies and avoids issues with a decline that is too rapid or too gradual [12]. The oxygen concentration two increments above the first signs of hypoxia stress (a change in ventilation rate, aquatic surface respiration (ASR) or loss of equilibrium) was selected as the upper boundary concentration (3.1 mg l^−1^). The oxygen treatments, 0.1 mg l^−1^ and 0.7 mg l^−1^, required 2.0 g and 0.5 g per 17.5 l treatment tank respectively of dissolved sodium sulphite in order to reach low DO levels. Sodium sulphite was added to treatment tanks as a solid and stirred prior to immersing fish within aquaria. The use of sodium sulphite in developing low DO for experimentation on fish is common [11], [16], [17] and although salt is not directly lethal to fish at the concentrations used here [18], the potential for compounding effects of increasing salinity and other toxic effects of the compound on hypoxia tolerance may warrant further investigation.

Experiments started by netting 10 fish and transferring them from 750 l holding tanks directly into an experimental treatment tank set to a pre-determined DO concentration. Immersing fish directly within experimental tanks set to a pre-determined DO concentration avoided bias associated with differences in time required to reduce oxygen levels among tanks but may have contributed to elevated rates of mortality at low oxygen levels. Water from holding tanks was used in all experimental and control tanks to avoid additional stress during transfer. Dissolved oxygen, temperature and pH were measured in each experimental and control tank at intervals of 0 h, 1 h, 4 h, 8 h, 16 h, 24 h, 32 h, 40 h, 48 h and DO concentrations were regulated with bubbled N_2_ gas or atmospheric air between intervals if necessary, to ensure treatment DO levels were maintained.

Mortality assessments were made during water quality testing intervals and death was classified as a loss of equilibrium and the cessation of gill ventilation. A binomial classification of dead (1) or alive (0) at the end of the 48 h experimental period was used in statistical analyses, although all mortalities were removed immediately from treatments and standard length (mm) and mass (±0.01 g) were measured. Aquatic surface respiration was quantified every five minutes for the first two hours of each experiment and then at four-hourly intervals up to 48 h. Aquatic surface respiration measurement periods typically lasted less than five seconds and classified fish as performing ASR (1) or alive but not performing ASR (0). Individuals ventilating with their mouths touching the top 1 mm of the water surface layer were recorded as performing ASR [12], whereas fish simply swimming near the surface were excluded. A viewing curtain prevented disturbing fish when ASR behaviour was recorded. Binomial ASR observations over the 48 hr experimental period were used in statistical analyses.

### Simulated hypoxic blackwater

A gradient of five simulated blackwater concentrations across aquaria was developed by immersing different masses of naturally abscised river red gum *Eucalyptus camaldulensis* leaf litter within experimental tanks. Oxygen concentrations were reduced via natural leaf litter decomposition and fish respiration within aquaria and did not require the use of nitrogen or sodium sulphite. Lethal oxygen concentration (LC_50_) and ASR_50_ measurements were otherwise conducted in accordance with hypoxic freshwater experiments. Leaf litter was collected from a fallen branch on private property along the Murray River between Albury and Howlong, NSW. A private land owner granted permission to collect samples and the NSW Office of Environment and Heritage confirmed that, within this region, the species is not threatened, or part of a threatened ecological community, and that no permit is required for scientific collection.

Prior to experimentation, leaf litter and twigs, ≤5 mm diameter were oven-dried at 60°C for 24 to 48 hrs. Five geometrically increasing masses of dried leaf litter containing 0 g (control), 3 g, 9 g, 27 g, and 81 g were placed in nylon stockings weighted with 250 g of cobble. These masses resulted in effective leaf litter concentrations ranging from 0 g l^−1^ to 4.59 g l^−1^ among aquaria which resulted in an oxygen concentration gradient ranging from 8.4±0.4 mg l^−1^ down to 0.46±0.28 mg l^−1^ respectively without the use of nitrogen or sodium sulfite. Controls consisted of cobble in nylon stockings but no leaf litter.

Leaf litter bags were allowed to soak for 24 hrs prior to experimentation and they were then removed and 10 fish were immersed in aquaria. Fish mortality, ASR assessments, and water quality measurements of DO, pH and temperature were carried out using the same methodology as described in hypoxic freshwater experiments. In addition, two 60 ml water samples were taken from each simulated blackwater treatment at the 0 h and 48 h interval. The mean concentration of DOC for each leaf litter treatment was used to examine its effect on fish mortality and ASR responses. Dissolved organic carbon concentrations were determined at a National Association of Testing Authorities (NATA) Australia accredited lab where an international standard method, 5310 C [19], was applied in which total organic carbon is considered all non-purgeable carbon.

### Natural blackwater event

Water quality conditions of experimental treatments were compared to a naturally occurring hypoxic blackwater event sampled in the MDB from the Edward River (Natural 1) and Wakool River (Natural 2) in December, 2010. John and Anthony Conallin from the Murray CMA and Luke Pearce from the NSW Department of Primary Industries confirmed the presence or absence of fish species mortalities in each river following the event. Water quality measurements were recorded during the hypoxic black water conditions, while fish mortalities in the field were verified during and after events. Water quality measurements of DO, pH and temperature were collected in flowing water within the main river channel during both events and water samples of less than one litre were collected for measurement of DOC concentration. Collection of water samples was made with permission from private land owners and the NSW Office of Water.

### Statistical analyses

Generalised Linear Mixed Models (GLMM's) [20] fit by maximum likelihood were used to test hypotheses and evaluate which combination of variables (DO, species, DOC, temperature, pH) best predicted mortality and ASR responses. Significance was evaluated at a 5% alpha level of type I error and results are presented as means ±1 SE unless otherwise stated. Observations of individual fish mortalities and ASR responses to low dissolved oxygen experiments were fitted to separate GLMM's using a binomial logit function in the ‘lme4’ package developed for R [20]. Individual responses of fish were nested randomly within tanks and we tested two null hypotheses: 1) there are no species-specific differences in lethal oxygen concentrations and ASR responses and 2) lethal oxygen concentrations and ASR responses are not influenced by increasing DOC in simulated blackwater.

Null hypotheses one and two were tested using the GLMM formula: Mortality or ASR∼DO+ species +DOC+ temperature +pH+(1|tank) where mortality  =  dead/1 or alive/0; ASR =  performing ASR/1 or alive but not performing ASR/0; DO =  mean DO within tank; species  =  species; DOC =  mean dissolved organic carbon concentration; temperature  =  mean temperature; pH =  mean pH and tank  =  experimental treatment aquaria set to a pre-determined DO concentration or simulated blackwater treatment. Null hypothesis one was rejected if species was a significant factor influencing mortality or ASR responses and null hypothesis two was rejected if DOC significantly affected either response. All possible combinations of factors were modelled and the Akaiki Information Criterion (AIC) was used to determine which model provided the most parsimonious fit to mortality and ASR responses. If correlation coefficients between fixed terms exceeded +/−0.70 then the term explaining the least amount of variation was excluded from the model. Residuals were checked visually for random dispersion and normality [20].

Lethal DO concentrations (LC_50's_) and aquatic surface respiration (ASR_50's_) values were estimated by fitting logistic regressions to individual responses of fish predicted by the most parsimonious GLMM for mortality and ASR data: 
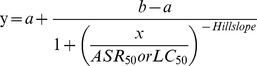
where the fitted parameter *a* is the minimum *y*-axis value, *b* is the asymptote, LC_50_ or ASR_50_ represent the median response and *hillslope* is the slope at its midpoint [21]. Logistic functions were restricted to data that met the criteria of normality and constant variance and parameters were reported only for models that provided significant (P<0.05) fits to the GLMM predicted values. Logistic regressions were fitted to the mean observed DO concentration measured within tanks rather than predetermined oxygen targets.

## Results

### Lethal oxygen concentrations

**Figure 2:**
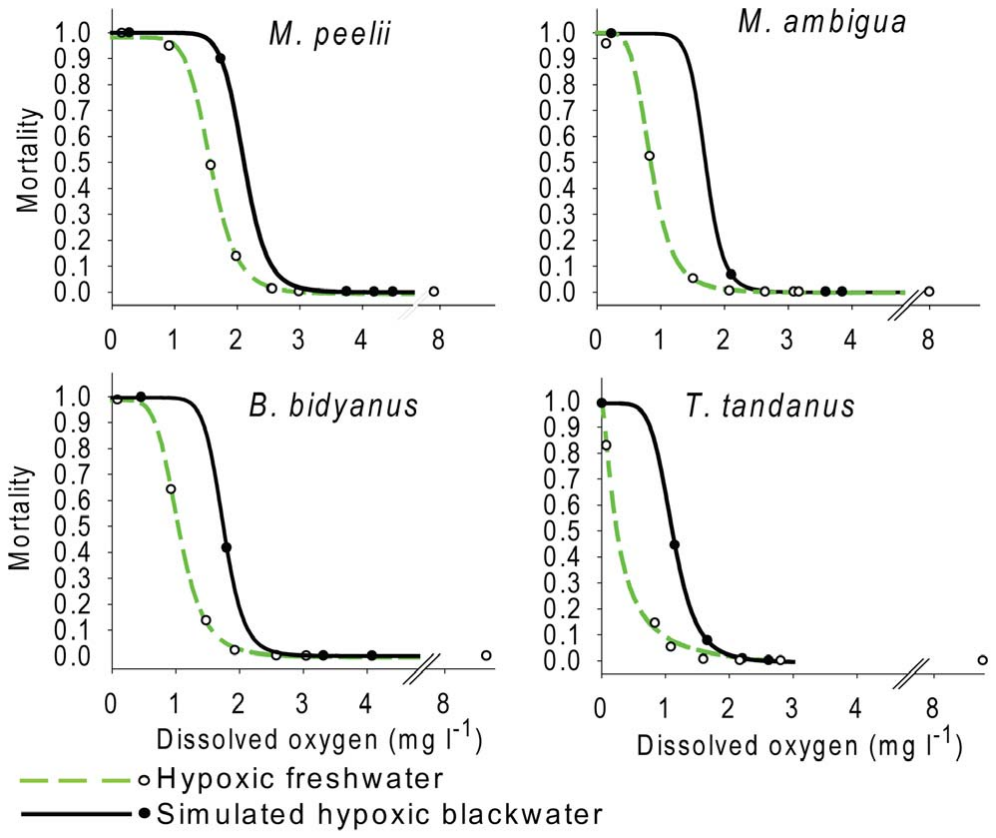
Lethal oxygen concentrations of four Murray-Darling Basin fishes. Proportion of juvenile Murray cod *Maccullochella peelii* (Mitchell), golden perch *Macquaria ambigua* (Richardson), silver perch *Bidyanus bidyanus* (Mitchell) and eel-tailed catfish *Tandanus tandanus* (Mitchell) dead at different dissolved oxygen concentrations at 25–26°C. Solid black and dashed green lines represent significant (*P*<0.05) logistic regressions fitted to simulated hypoxic blackwater and hypoxic freshwater experiments respectively. Circles represent model predicted values.

There were significant species-specific differences in mortality responses to low DO (Table 2) and therefore null hypothesis one was rejected. The GLMM factors: DO, species and DOC provided the most parsimonious (AIC = 168) fit to mortality responses and estimated values from this model were fitted significantly by logistic regression in all species (Figure 2; Table 3). Species-specific differences in sensitivity to low DO were similar in both hypoxic freshwater and simulated blackwater experiments (Table 3). The most tolerant species in hypoxic freshwater and simulated hypoxic blackwater was *T. tandanus* and the most sensitive was *M. peelii* (Figure 2). The oxygen concentration which caused 50% mortality in *M. peelii* within hypoxic freshwater experiments resulted in 4%, 9% and 4% percent mortality in *M. ambigua*, *B. bidyanus* and *T. tandanu*s respectively. A repeated treatment for *B. bidyanus* could not be conducted due to a limited number of fish available although the standard error of LC_50_ (Table 3) estimates for this species was the same or lower than other species in hypoxic freshwater and simulated blackwater experiments.

**Table 2:** Generalised Linear Mixed Model (GLMM) estimates ±SE combining all factors to predict mortality and aquatic surface respiration (ASR) responses of four Murray-Darling Basin fishes exposed to hypoxic freshwater and simulated blackwater.

	Mortality				ASR			
Factors	Standardized estimate	SE	*z* value	*P*-value	Standardized estimate	SE	*z* value	*P*-value
Temperature (°C)	0.31	0.21	1.45	NS	−0.04	0.22	−0.18	NS
pH	0.56	0.22	2.57	<0.05	0.99	0.25	4.01	<0.001
Dissolved organic carbon	0.28	0.06	4.78	<0.001	−0.06	0.02	−4.72	<0.001
Dissolved oxygen	−4.22	0.53	−7.87	<0.001	−1.51	0.18	−8.47	<0.001
Murray cod *Maccullochella peelii*	4.8	0.74	6.45	<0.001	1.50	0.41	3.63	<0.001
Golden perch *Macquaria ambigua*	1.72	0.63	2.71	<0.05	1.34	0.42	3.16	<0.05
Silver perch *Bidyanus bidyanus*	2.65	0.6	4.44	<0.001	1.24	0.41	3.06	<0.05
Eel-tailed catfish *Tandanus tandanus*	1.85	0.53	3.48	<0.001	0.40	0.38	1.04	NS
AIC	177				364			

*P*-values listed as NS represent no significant effect. The most parsimonious GLMM models according to the Akaiki Information Criterion (AIC) included: Dissolved Oxygen, Species and Dissolved Organic Carbon for mortality (AIC = 168) and ASR (AIC = 360) responses.

**Table 3:** Logistic function parameters predicting lethal concentrations (LC) of dissolved oxygen (DO) in four Murray-Darling Basin fishes.

	Hypoxic freshwater					Simulated hypoxic blackwater				
Species	*LC_50%_* DO (mg l^−1^)	*a*	*b*	*Hill slope*	*P-value*	*LC_50%_* DO (mg l^−1^)	*a*	*b*	*Hill slope*	*P-value*
Murray cod *Maccullochella peelii*	1.58 (<0.01)	<−0.01 (<0.01)	0.98 (<0.01)	7.65 (0.13)	P<0.001	2.11 (<0.01)	<−0.01 (<0.01)	0.99 (<0.01)	11.41 (0.01)	P<0.001
Golden perch *Macquaria ambigua*	0.85 (<0.01)	<−0.01 (<0.01)	1.00 (0.01)	4.99 (0.19)	P<0.001	1.70 (<0.01)	<−0.01 (0.03)	0.99 (<0.01)	12.28 (0.03)	P<0.001
Eel-tailed catfish *Tandanus tandanus*	0.25 (0.06)	−0.03 (<0.01)	1.00 (0.10)	1.44 (0.21)	P<0.001	1.11 (<0.01)	<−0.01 (<0.01)	0.99 (<0.01)	5.99 (0.02)	P<0.001
Silver perch *Bidyanus bidyanus*	1.04 (<0.01)	<−0.01 (<0.01)	0.99 (<0.01)	5.32 (0.04)	P<0.001	1.75 (<0.01)	<−0.01 (<0.01)	1.00 (<0.01)	10.91 (0.01)	P<0.001

Experiments were conducted over 48 hrs at 25–26°C in hypoxic freshwater and simulated hypoxic blackwater. Parameters were estimated ± (SE) for significant (*P*<0.05) logistic regressions.

Increasing DOC concentrations from simulated blackwater significantly affected mortality (Table 2) and therefore null hypothesis two rejected. Reductions in pH also significantly affected mortality but due to autocorrelation with DOC, its effect reported in Table 2 was modelled as an independent factor. Estimates of LC_50's_ (Table 3) for all species occurred at higher oxygen concentrations in the presence of elevated DOC and lower pH's that occurred in simulated blackwater experiments (Table 1; 4). The oxygen concentration which caused more than 50% mortality in all species under simulated blackwater conditions resulted in less than 10% mortality of all species within hypoxic freshwater (Figure 2).

**Table 4:** Mortality (%) of four Murray-Darling Basin fishes exposed to simulated blackwater experiments and confirmed mortalities associated with natural hypoxic blackwater events in the Edward-Wakool river system in 2010/11.

Blackwater sample	DO (mg l^−1^)	pH	Temp. (°C)	DOC (mg l^−1^)	Mortality (%)			
					Murray cod *Maccullochella peelii*	Golden perch *Macquaria ambigua*	Eel-tailed catfish *Tandanus tandanus* ^a^	Silver perch *Bidyanus bidyanus*
Natural 1	1.07 (0.08)	4.18 (0.04)	19.4 (0.02)	17.7 (0.08)	Confirmed	Confirmed	*-*	Confirmed
Natural 2	1.98 (0.11)	6.25 (0.09)	18.7 (0.05)	16.1 (0.07)	Confirmed	Confirmed	-	Confirmed
Control (0 g l^−1^)	8.4 (0.4)	7.41 (0.05)	25.62 (0.03)	2.7 (0.05)	0	0	0	0
Simulated blackwater (0.17 g l^−1^)	4.83 (0.38)	7.22 (0.04)	26.26 (0.08)	3.58 (0.38)	0	0	0	0
Simulated blackwater (0.51 g l^−1^)	4.0 (0.3)	7.05 (0.01)	26.09 (0.06)	4.93 (0.13)	0	0	0	0
Simulated blackwater (1.53 g l^−1^)	1.8 (0.44)	6.76 (0.03)	25.93 (0.06)	9.45 (0.55)	90	7	8	45
Simulated blackwater (4.59 g l^−1^)	0.46 (0.28)	6.05 (0.01)	26.41 (0.05)	30.0 (0.7)	>99	>99	>99	>99

Water quality and dissolved organic carbon (DOC) measurements of simulated blackwater treatments (mass of leaf litter g l^−1^) were aggregated across species for comparison with two natural blackwater events. Mean ±SE.

a. Historically present in the Edward-Wakool system but not sampled in recent unpublished fisheries surveys.

Time to mortality increased with increasing oxygen concentration from 15.39±1.68 min. (mean ±*SE*) at 0.1± mg l^−1^ DO to 128.60±32.61 min. at 0.7 mg l^−1^. Logistic regressions in simulated blackwater experiments (Figure 2) predicted that first mortalities may start at oxygen concentrations as high as 2.4 mg l^−1^ to 3.1 mg l^−1^ in *T. tandanus* and *M. peelii* respectively. First mortalities of *B. bidyanus* and *M. ambigua* in hypoxic blackwater were predicted to start at 2.8 mg l^−1^ and 2.6 mg l^−1^ respectively. Several deaths of *B. bidyanus* and *M. peelii* were attributed to aggressive behaviour recorded in one control and the 0.17 g l^−1^ leaf litter treatment. Evaluating aggressive behaviour in relation to DO was beyond the scope of this experiment but it is interesting to note that this activity was limited to oxygen concentrations exceeding 4.45 mg l^−1^ and was most common above 8 mg l^−1^. The effect of aggressive behaviour on mortality responses was minimized by the application of a GLMM which predicted that no mortalities could be attributed statistically to oxygen limitation at such high concentrations.

Natural blackwater events resulted in verified deaths of adult *M. peelli, M. ambigua* and *B. bidyanus* in the Edward-Wakool River system (Table 4). The natural blackwater events occurred at lower water temperatures but were associated with more acidic pH's than the simulated blackwater treatments in the present study. The dissolved oxygen concentration, pH and DOC of natural blackwater events fell between the conditions of the 1.53 g l^−1^ and 4.59 g l^−1^ simulated blackwater treatments in the present study. Percent mortality of *M. peelii* was the highest of all species and ranged from 90% in the 1.53 g l^−1^ to 99% in the 4.59 g l^−1^ simulated blackwater treatments (Table 4). Natural blackwater events resulted in confirmed mortalities of all species except for *T. tandanus* which was historically present in the Edward-Wakool system but has not been sampled in recent unpublished fisheries surveys (J. Conallin personal communication). *Tandanus tandanus* also had the highest tolerance to low DO in simulated blackwater treatments (Table 3) and field measurements of DO during the natural blackwater events did not reach the LC_50_ estimates for this species.

### Aquatic surface respiration

**Figure 3:**
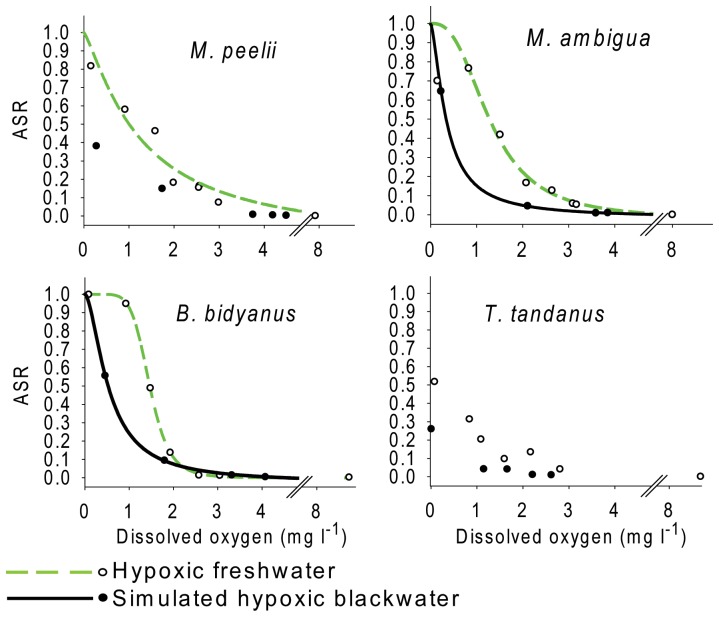
Aquatic Surface Respiration (ASR) in four Murray-Darling Basin fishes. Proportion of juvenile Murray cod *Maccullochella peelii* (Mitchell), golden perch *Macquaria ambigua* (Richardson), silver perch *Bidyanus bidyanus* (Mitchell) and eel-tailed catfish *Tandanus tandanus* (Mitchell) performing ASR at different dissolved oxygen concentrations at 25–26°C. Solid black and dashed green lines represent significant (*P*<0.05) logistic regressions fitted to simulated hypoxic blackwater and hypoxic freshwater experiments respectively. Circles represent model predicted values.

Aquatic surface respiration was reported for the first time in juveniles of all four species and this behaviour was performed in hypoxic freshwater and simulated hypoxic blackwater experiments (Figure 3). Field observations of adult *M. peelii* swimming with their body positioned upward and their mouth at the water surface (Figure 1) were recorded in the Edward River during a hypoxic blackwater event. Null hypothesis one was rejected on the basis of significant differences in ASR responses among species (Table 3). The GLMM factors: DO, species and DOC provided the most parsimonious (AIC = 360) fit to ASR data, although pH was also a significant factor influencing the response (Table 3). Estimated values from the most parsimonious GLMM were fitted by a logistic regression for all species except *M. peelii* in the blackwater simulation and both experiments on *T. tandanus* (Table 5). In both species where the model did not fit, the logistic regression failed to predict ASR responses because a majority of individuals did not perform this behaviour at the lowest oxygen concentrations (Figure 3).

**Table 5:** Logistic function parameters predicting the proportion of live fish performing aquatic surface respiration (ASR) over 48 hrs at different dissolved oxygen (DO) concentrations.

	Hypoxic freshwater					Simulated hypoxic blackwater				
Species	*ASR_50%_* DO (mg l^−1^)	*a*	*b*	*Hill slope*	*P-value*	*ASR_50%_* DO (mg l^−1^)	*a*	*b*	*Hill slope*	*P-value*
Murray cod *Maccullochella peelii*	1.25 (0.11)	−0.15 (0.06)	1.00 (0.05)	1.25 (0.11)	P<0.001	-	-	-	-	NS
Golden perch *Macquaria ambigua*	1.31 (0.07)	−0.03 (0.04)	1.00 (0.04)	2.67 (0.34)	P<0.001	0.35 (0.10)	−0.02 (<0.01)	1.00 (0.17)	1.53 (0.16)	P<0.001
Eel-tailed catfish *Tandanus tandanus*	-	-	-	-	NS	-	-	-	-	NS
Silver perch *Bidyanus bidyanus*	1.47 (<0.01)	<−0.01 (<0.01)	1.00 (<0.01)	6.66 (0.06)	P<0.001	0.54 (0.13)	−0.03 (0.01)	1.00 (0.20)	1.68 (0.20)	P<0.001

Experiments were conducted at 25–26°C in hypoxic freshwater and simulated hypoxic blackwater. Parameters were estimated ± (SE) for significant (*P*<0.05) logistic regressions.

Null hypothesis two was rejected because ASR responses were influenced significantly by the presence DOC (Table 3) under simulated blackwater conditions. Proportionally fewer individuals of all species performed ASR under blackwater simulations when compared to hypoxic freshwater experiments (Figure 3). First observations of ASR behaviour (Figure 3) usually preceded first observations of mortality within species (Figure 2). Logistic regressions in hypoxic freshwater predicted that ASR may start at oxygen concentrations ranging from 2.59 mg l^−1^ to 4.96 mg l^−1^ in *B. bidyanus* and *M. peelii*, although first observations in these species occurred at 1.93 mg l^−1^ and 2.99 mg l^−1^ respectively. The first observation of ASR in *T. tandanus* and *M. ambigua* occurred in hypoxic freshwater at 2.81 mg l^−1^ and 3.17 mg l^−1^ respectively.

The proportion of fish performing ASR increased in all species and experiments with declining dissolved oxygen concentration. All species performed ASR (Figure 3) at levels of dissolved oxygen where no mortality was recorded (Figure 2) and at more extreme levels of hypoxia where mortality ensued. It is notable that a single *M. ambigua* survived the entire 48 h period at 0.15 mg l^−1^ DO and continuously utilised ASR behaviour. At the same oxygen concentration total mortality was observed across all species and other individuals of the same species within less than 60 min.

## Discussion

### Hypoxia thresholds

The fishes examined in this study were unexceptional in their ability to survive in low dissolved oxygen freshwater and in simulated blackwater. Significant species-specific differences were apparent, although rates of mortality increased following a logistic function when oxygen concentrations fell below a threshold of approximately 2–3 mg l^−1^ at 25–26°C. The range of hypoxia tolerances reported for juvenile lowland river fishes here may be considered intermediate on a global-scale comparison with other freshwater fishes. On the extreme end of the hypoxia tolerance spectrum, the crucian carp *Carassius carassius* (L.) and goldfish *Carassius auratus* (L.) can withstand anoxia at temperatures of 20–25°C for a few hours or days [22], whereas exposing some cold-adapted fishes to oxygen saturated (>8 mg l^−1^) water at 25°C will result in mortality [23].

The hypoxia tolerances of the freshwater fishes examined here were comparable to other temperate stream fishes world-wide [24]–[26], whereby *T. tandanus* was the most tolerant and *M. peelii* amongst the most sensitive. The median lethal oxygen concentrations observed here ranged from 0.25 mg l^−1^ to 1.58 mg l^−1^ at 25–26°C in freshwater and 1.11 mg l^−1^ to 2.11 mg l^−1^ in simulated blackwater. At 25°C in freshwater the LC_50's_ of 35 fishes in the mid-western United States ranged from 0.49 mg l^−1^ to 1.59 mg l^−1^ [24]. At the same temperature, the mean dissolved oxygen concentration resulting in a loss of equilibrium ranged from 0.95 mg l^−1^ to 2.66 mg l^−1^ in other prairie stream fishes in the United States [25]. The LC_50's_ of four native New Zealand fishes and rainbow trout, *Oncorhynchus mykiss* (Walbaum) ranged from 0.54 mg l^−1^ to 2.65 mg l^−1^ at 15°C [26].

It is not appropriate to extend our experimental estimates to predict the magnitude of hypoxic blackwater fish kills in the wild, although our study provides some indication as to whether water quality conditions are approaching lethal thresholds. Notwithstanding the considerable physiological importance of cooler water conditions on hypoxia tolerance [23], which prevailed during the natural blackwater events in 2010/11 [2]. Our results suggest that low oxygen levels of less than 2.0 mg l−1, in addition to the high DOC (>10 mg l^−1^) and low pH (4.2–6.3), which occurred in the Edward-Wakool system and across much of the southern basin for several weeks or months during these events [2], must have approached or exceeded lethal threshold limits for all species except for *T. tandanus*. Confirmed field mortalities of the remaining three species in the Edward-Wakool system indeed corroborate this assumption. It is important to consider, however, that fish in the wild may be able to avoid hypoxic blackwater by seeking refuge habitat [6], while fish in our study were confined within experimental treatment tanks that may have exaggerated estimates of mortality.

Our estimates may have been exaggerated by not gradually acclimating fish to hypoxia. Lethal hypoxia experiments on freshwater fish in the United States suggest that metabolic rates require 24 hours to return to standard levels after handling [27]. We suggest, therefore, that future studies focusing on lethal hypoxia thresholds in fish utilize a gradual reduction in oxygen levels following a minimum recovery period of 24 hours after handling. Conversely, our lethal oxygen concentrations may have underestimated rates of mortality in the wild given that our experiments were brief (48 hrs) compared to the weeks or months [2] of hypoxic blackwater conditions that fish may be exposed to in the wild.

Hypoxia is a natural phenomenon in aquatic ecosystems, but conservation and management mitigation measures may be required if the frequency, duration, spatial extent or potency of these events has changed due to anthropogenic pressures [2]. Low oxygen survival and sub-lethal thresholds, including but not limited to those reported here, could be developed into hypoxia monitoring and warning systems that catalyse fish kill mitigation approaches and reporting [7]. The thresholds reported here provide a starting point to begin developing hypoxia mitigation strategies but must be carefully interpreted in light of potential sub-lethal effects [6], ambient and antecedent environmental conditions. It is essential to consider that hypoxia thresholds will change according to temperature, pH, DOC [4], [10] and other water chemistry parameters. Consequently, a better understanding how water quality parameters interact with fish physiology will be essential in developing future hypoxia mitigation plans. Elevated rates of mortality in blackwater simulations in the present study, when compared to low oxygen freshwater conditions, demonstrate that small reductions in pH and elevated DOC can exacerbate hypoxia driven mortality. Reductions in pH decrease the oxygen affinity of gills and it therefore directly affects hypoxia tolerance. The reduced pH of natural blackwater events and blackwater simulations is most likely attributable to increasing concentrations of dissolved organic acids, carbon dioxide or toxic polyphenols [4]. Polyphenols make up a large component of DOC in Australian rivers and at high concentrations some of these compounds are lethal to fish [4], [10].

Future research focusing on temperature effects on hypoxia-driven mortality and sub-lethal effects on MDB fishes is warranted. Since hypoxia-driven mortality declines exponentially with decreasing temperature [23], [28], the oxygen thresholds reported in this study could be used as precautionary management reference points under similar water quality conditions and temperatures equal to or below 25–26°C. Rising temperature directly increases metabolic demands in ectotherms [29] and therefore it is reasonable to assume for our study species that lethal oxygen concentrations will increase, or decrease, according to the ambient thermal conditions. Resting oxygen metabolism in species of fish generally increase by a factor of approximately 2.4 for every 10°C (Q_10_) temperature interval between 0°C and 30°C [29]. The corresponding Q_10_ for hypoxia tolerance has been estimated to be 2.09 for marine fish [28], although comparable hypoxia Q_10_ estimates for freshwater fish assemblages do not exist. Temperature was held constant in the present study to control for its effect, although future research on hypoxia tolerances of MDB fishes conducted at a range of temperatures is needed.

All four species performed ASR which is an adaptation that many fishes exhibit when exposed to low dissolved oxygen [6], [16]. It is notable that ASR has not previously been documented in the peer-reviewed literature for any of the species examined here. The ASR_50's_ reported here were within the upper range of 0.48 to 1.98 mg l^−1^ [12] at 25°C for small-bodied native MDB fishes including Australian smelt *Retropinna semoni* (Weber), flat-headed galaxias *Galaxias rostratus* (Klunzinger), carp gudgeon compex *Hypseleotris spp*. and southern pygmy perch *Nannoperca australis* (Gunther). An unexpected observation from our experiment was that ASR was performed less frequently under simulated blackwater conditions than in hypoxic freshwater conditions. It is possible that a reduction in ASR behaviour within blackwater simulations may have resulted as a consequence of fish experiencing other physiological stressors, including toxic polyphenols [4] or reduced pH, beyond the direct effects of hypoxia. Nonetheless, field observations of *M. peelii* performing ASR (Figure 1) under more acidic pH's and higher DOC concentrations than were simulated here (Table 4) suggest that this behaviour is utilized by adults when exposed to hypoxic blackwater conditions in the wild.

### Life-history and hypoxia tolerance

The species-specific differences in hypoxia tolerance observed here were broadly consistent with expectations based on known life-history strategies and evolutionary lineages of the species examined. For example, a species of catfish (*T. tandanus*) that typically occupies benthic slow flowing habitats, including wetlands and floodplains, and is relatively sedentary [30] compared to the other fishes studied was the most hypoxia and blackwater tolerant fish examined. The high tolerance of this species is typical of Siluriform fishes and, although not observed in *T. tandanus*, some species in this order are capable of aerial respiration [31]. Although the most hypoxia tolerant species (*T. tandanus*) also had the greatest mean body mass and length within our experiments, species-specific differences in hypoxia tolerance among the remaining species followed no discernable pattern associated with body mass. *Macquaria ambigua* (Percichthyidae) and *B. bidyanus* (Terapontidae) which are more mobile but spawn in potentially low oxygen floodplain environments [30] were the second most tolerant group to low dissolved oxygen and blackwater.

One outstanding question remaining from the present study is whether or not the hypoxia tolerances of the juveniles examined here are representative of adults that are more commonly reported in fish kills in the MDB [8], [9]. A review on this subject across multiple species of marine fish concluded that body size itself plays little or no direct role in determining hypoxia tolerance and that the tolerance of a given species is usually size-independent after the larval stage [32]. The authors of this study purport that if there are size-specific differences in hypoxia tolerance, larger individuals of a given species would generally be expected to survive longer periods of time under hypoxic conditions compared to smaller individuals. However, there are examples of size, trophic status and developmental stage-dependent hypoxia tolerances within and among species of fish [23], [33], [34]. Therefore, further research focusing on hypoxia tolerances of different developmental stages, body sizes and life-histories of MDB fishes would be useful. Mortality of *M. ambigua* larvae was identified by [4] in response to dissolved oxygen concentrations as high as 4.3 mg l^−1^at 24.5°C which suggests that larvae may be more sensitive than the juveniles examined here. Our results do not preclude the possibility that adults may be more, or less, sensitive to hypoxia than juveniles but emphasizes that *M. peelii* appears to be less hypoxia tolerant than other fishes in the MDB and elsewhere world-wide [24]–[26]. The relatively high hypoxia sensitivity of *M. peelii* may also explain, in part, why this species is disproportionately reported in blackwater fish kills in the MDB [7], [8], [9].

In contrast to popular anecdote, it appears that the hypoxia sensitivity of *M. peelii* may not be entirely related to its large maximum attainable body mass (>100,000 g). The *M. peelii* examined here were juveniles (<1.2 g) and not the largest species yet they were still the most sensitive examined. The comparatively low hypoxia tolerance of *M. peelii* appears to be species-specific or, at least, not entirely size-specific. We suggest that Australia's largest freshwater fish species is sensitive to hypoxia, not because it a large-bodied apex predator *per se*, but because it has evolved a life-history strategy that primarily utilizes lotic river channel environments and habitats [35], [36], which are less prone to severe hypoxic blackwater conditions than floodplains, wetlands or backwater areas commonly occupied by other species [32]. It is notable, however, that juveniles of all four of the species examined here were historically stranded in large numbers within isolated floodplain pools following spring over bank floods [37]. Similar scale floodplain strandings of juveniles of large-bodied species have rarely, if ever, been reported in recent years within the MDB. Small-bodied and invasive species are now commonly found in isolated floodplain pools of the MDB and these fishes are seemingly unaffected by extremely high DOC (16–50 mg l^−1^) and low dissolved oxygen (0.4–6.8 mg l^−1^) at temperatures ranging from 15–18°C [10].

The moderate hypoxia survival thresholds reported for the species examined here are in stark contrast to the prevalence of unique adaptations, such as air-breathing and other physiological mechanisms, that facilitate long-term survival of fish in blackwater rivers [38], [39]. Adaptations to survive prolonged and widespread hypoxia are obligatory in many blackwater rivers, while fishes of the MDB may have historically been able to withstand blackwater events that were more localized and may have occurred under cooler winter conditions [2], without anthropogenic barriers that could limit access to refuge habitat. For comparison, floodplain forests surrounding Amazonian blackwater rivers are inundated each year for three to eleven months, covering an area of approximately 70,000 km^2^ [39], whereas blackwater events in the MDB are typically localized at the river or wetland scale and are characterised by extreme variability in area, timing and duration [1], [3]. There is evidence, however, that the severity of hypoxic blackwater events may be increasing due to anthropogenic pressures [2] and results from our study suggest that fish which have life-histories adapted to in-channel environments [35], such as *M. peelii*, may be most vulnerable.
